# CAREGIVING INTENSITY AND LABOR FORCE PARTICIPATION AMONG CAREGIVERS: THE ROLES OF INCOME AND PAID HELP

**DOI:** 10.1093/geroni/igae098.2738

**Published:** 2024-12-31

**Authors:** Shan Qu, Jeffrey Burr

**Affiliations:** University of Massachusetts Boston, Boston, Massachusetts, United States; University of Massachusetts Boston, Boston, Massachusetts, United States

## Abstract

Framed within Role Theory, this study examined the association between caregiving intensity and labor force participation among caregivers and whether income and paid help moderated this relationship. We used data from the National Health and Aging Trends Study and the National Study of Caregiving. Caregiving intensity was measured by days and hours of caregiving in the last month. Care was considered high-intensity if a respondent provided an average of 21 hours or more of care per month. Low-intensity caregivers provided 20 hours of care or less per month. Labor force participation was divided into three categories: ‘1=unemployed/retired,’ ‘2=worked less than 35 hours,’ and ‘3=worked 35 hours or more.’ Compared to those with low-intensity caregiving, high-intensity caregivers had a lower likelihood of working part-time than those who were not working. Compared to not having a paid helper, having a paid helper was associated with an increased likelihood of working part-time and full-time compared to those who were not working. As household income increased, the likelihood of being a part-time worker and the likelihood of being a full-time worker increased. The moderation model results showed the likelihood of working part-time compared to being unemployed or retired was lower when caregiving intensity was higher. No other moderation effect was found for paid work. In addition, the likelihood of working part-time or full-time increased as annual income increased. We believe the findings contribute to policies that increase access to paid help and may ultimately assist caregivers to remain active in the job market.

